# Familial, Social, and Individual Factors Contributing to Risk for Adolescent Substance Use

**DOI:** 10.1155/2013/579310

**Published:** 2013-03-20

**Authors:** Mackenzie Whitesell, Annette Bachand, Jennifer Peel, Mark Brown

**Affiliations:** ^1^Department of Environmental and Radiological Health Sciences, Colorado State University, Fort Collins, CO 80523, USA; ^2^Department of Clinical Sciences, Colorado State University, Fort Collins, CO 80523, USA; ^3^Department of Ethnic Studies, Colorado State University, Fort Collins, CO 80523, USA

## Abstract

Data from the National Institute on Drug Abuse (NIDA) and the Centers for Disease Control and Prevention (CDC) reveal high numbers of adolescent substance use in the United States. Substance use among adolescents can lead to increased risk of transmission of sexually transmitted infections, vehicular fatalities, juvenile delinquency, and other problems associated with physical and mental health. Adolescents are particularly susceptible to involvement in substance use due to the underdeveloped state of the adolescent brain, which can lead to reduced decision-making ability and increased long-term effects of drugs and alcohol. Understanding the causes of adolescent substance use is vital for successful prevention and intervention programs.

## 1. Introduction

NIDA, part of the National Institutes of Health (NIH), has continuously monitored trends of substance use among adolescents through providing funding to Monitoring the Future (MTF) Project since 1975 [1]. MTF consists of a series of questionnaires that are administered to adolescents throughout the country in the 8th, 10th, and 12th grades regarding their beliefs and practices relating to substance use [1]. Though the sample population evaluated is restricted in its size (*n* = 50,000), is limited to adolescents attending school, and relies on self-reported behavior, the statistics generated by the MTF project provide a framework for understanding trends in adolescent substance use in the United States [1].

The 2011 MTF data reveal current predominant issues regarding adolescent substance use. The prevalence of cigarette use and binge drinking, defined by NIDA as five or more consecutive drinks in the past two weeks, has decreased over the past five years [2]. However, use of tobacco products remains high, with 2.4% of 8th graders, 5.5% of 10th graders, and 10.3% of 12th graders smoking every day [2]. Similarly, binge drinking was reported by 6.4% of 8th graders, 14.7% of 10th graders, and 21.6% of high school seniors [2].

The past five years have also been marked by an increase in marijuana use, with 12.5% of 8th graders, 28.8% of 10th graders, and 36.4% of 12th graders reporting use in the last year on the national 2011 MTF survey, which NIDA associates with decreases in perceived risk of harm [2]. The CDC found in their 2009 Youth Risk Behavioral Surveillance Survey (YRBSS) that 36.8% of high school students had used marijuana at some point in their life and that 20.8% had used it during the 30 days prior to the survey [3].

An emerging trend of adolescent substance use is the use of synthetic marijuana (commonly referred to as K2 or “spice”) [2, 4, 5]. Synthetic marijuana, a substance that generates parallel effects to marijuana, became popular in 2009, and in early 2011 it was temporarily banned for at least a year by the Drug Enforcement Administration (DEA) until a permanent ban can be enacted [4, 5]. In 2011, the national reported use of synthetic marijuana within the previous year was 11.4% among high school seniors [2].

Another key area of concern is use of prescription and over-the-counter (OTC) drugs for nonmedical purposes [2, 3]. The CDC reported that, in 2009, 20.2% of high school students had misused a prescription drug [3]. Commonly abused prescription drugs include Vicodin, Oxycontin, Adderall, and Ritalin, with the last two primarily prescribed for treatment of attention deficit hyperactivity disorder (ADHD) [3, 6]. Other substances commonly used for nonmedical purposes are tranquilizers and cough medicine [7]. NIDA reports that prescription and OTC drugs are the most commonly abused illicit substances among 12th graders [7].

These patterns of drug use among adolescents are associated with ramifications such as the spread of HIV/AIDS, increased risk for vehicular fatalities, and behaviors leading to juvenile delinquency. Substance use increases the risk of contracting HIV due to the potential for sharing needles and participating in risky behaviors [8]. It also aggravates health issues associated with HIV/AIDS [8]. Substance use is also a cause of dangerous driving in that it affects reaction time and judgment capabilities, particularly among teens aged sixteen through nineteen [9]. The 2009 YRBSS found that, in the 30 days prior to the survey, 28.3% of high school students nationwide rode in a vehicle whose driver had been drinking alcohol [3]. Furthermore, apart from being an illicit activity in and of itself, substance use is a risk factor for additional acts of juvenile delinquency, with substance use disorders among the most commonly diagnosed disorders within the juvenile justice system [10, 11].

Throughout this paper, the terms substance dependence and substance abuse will commonly be addressed. As defined by the fourth edition of the Diagnostic and Statistical Manual of Mental Disorders (DSM-IV), both substance dependence and substance abuse are classified as substance use disorders [12]. Substance Abuse is characterized by a pattern of substance use leading to neglect of roles or commitments, physical hazards, legal issues, or interpersonal problems [12]. Substance dependence is a more extreme diagnosis wherein use of the substance is reoccurring to the point of reducing important social and occupational activities, causing tolerance of the substance and/or withdrawal symptoms and causing the user to devote a great deal of time to obtain and use the substance [12]. Substance abuse and dependence can lead to other forms of mental disorders, including mood, anxiety, and sleep disorders [12].

Analyzing adolescent substance use and substance use disorder trends in the United States is vital in addressing public health concerns such as transmission of sexually transmitted infections (STIs), vehicular deaths, and further acts of juvenile delinquency. As new trends emerge, risk factors leading to substance use and substance use disorders must be continuously assessed in order to design and enact effective prevention and intervention programs. The purpose of this paper is to synthesize current research regarding risk factors that lead to substance use and substance use disorders among adolescents. From an epidemiological perspective, substance use will be viewed as an outcome (in place of, e.g., an infectious disease) made more probable by certain risk factors.

Familial, social, and individual risk factors will be addressed, and the way in which brain development may connect these factors to the outcome of substance use will be discussed. Risk factors categorized as familial include childhood maltreatment (abuse and neglect), familial substance abuse, and parent-child relationships. Social risk factors include association with deviant peers, popularity, bullying, and gang affiliation. Individual risk factors include ADHD and depression. These categories of risk factors (familial, social, and individual) are defined here for the purposes of this paper, but categorization of risk factors varies between researchers depending on the focus of the discipline.

## 2. Background

### 2.1. Cognitive Development

In terms of origins for risk factors, the effects on cognitive development represent a key component when addressing the issue of adolescent substance use. Because the brain undergoes considerable development during adolescence, this period is a time of increased vulnerability to stress and risk-seeking behaviors [13–15]. Stressful circumstances, including familial or social tensions and maltreatment, that occur during critical periods can cause increased reactivity to addictive drugs and thus heighten the potential for a substance use disorder to occur [16, 17].

As it is related to brain development, the adolescent period is characterized by decreases in grey matter, increases in white matter, increased dopaminergic connections feeding into the prefrontal cortex, and development of the limbic system [15]. Grey matter is the term used for neuron cells in the brain that participate in specialized functions of the brain, whereas white matter describes tissue containing axons that are responsible for the passage of information from the sensory organs to the cerebral cortex and participate in functions such as emotion and hormone regulation [18]. Some variations between genders has been noted with regard to brain development. Specifically, male adolescents tend to show more drastic decreases in grey matter and increases in white matter throughout adolescence [15]. This may be attributable to differences in sex hormones, which are released in high concentration during puberty [15, 19].

The prefrontal cortex is a region of the brain that is involved in processing and decision-making based on memory and reward assessment [14]. It responds to stimulus by dopamine, a neurotransmitter that has a critical role in pleasure and reward, as well as emotional responsiveness [18]. During adolescence, the prefrontal cortex undergoes significant development, including changes in thickness, dendritic structure, and increased growth of dopaminergic fibers [13, 20].

Another area of the brain that undergoes significant changes during adolescence is the limbic system [13]. The limbic system is a region of the brain that encompasses the hypothalamus, amygdala, hippocampus, and the nucleus accumbens [13, 21]. Functions of the limbic system include memory, emotional response, motivation, and reinforcing behaviors, all of which can play a role in repetitive patterns of substance use [21]. Most notably, the nucleus accumbens responds to dopamine when generating pleasure responses and reward cycles [22, 23].

The majority of addicting drugs, including nicotine, opiates, ethanol, and stimulants, increase the release of dopamine in humans [23, 24]. Increased release of dopamine can cause addiction to a substance because, in the presence of abnormally high amounts of dopamine, the brain begins to adjust by decreasing its natural production of the neurotransmitter [25]. Therefore, in order to maintain normal levels, the brain becomes dependent on a drug to supply dopamine production [25].

This addictive characteristic of dopamine in combination with its increased role in the development of the brain makes adolescence a critical period in developing the reinforcing behavior of substance use [15]. The process of development of the prefrontal cortex and limbic system during adolescence increases the likelihood that behaviors initiated during this vulnerable period will have long-term effects and continue throughout the lifespan [13, 26]. Furthermore, because the adolescent prefrontal cortex is not fully developed, it is characterized by increased sensitivity to reward and decreased ability to inhibit responses to stimulus, thus limiting decision-making ability and behavior control throughout adolescence [14, 26]. This trait may explain the proclivity towards risk-seeking behavior, such as substance use, that has been repeatedly shown to exist among adolescents [13, 15, 27].

## 3. Risk Factors for Adolescent Substance Use

There are an extensive number of risk factors that may contribute to the onset of substance use among adolescents. Herein, selected risk factors for adolescent substance use are divided into three primary categories: familial, social, and individual. A comprehensive review of all risk factors is not practical with the scope of this paper. Thus, for the purpose of this paper, the most common and serious risk factors have been highlighted.

### 3.1. Familial Risk Factors

Familial risk factors include childhood maltreatment (including abuse and neglect), parental or familial substance abuse, marital status of parents, level of parental education, parent-child relationships, familial socioeconomic status, and child perception that parents approve of their substance use. Child maltreatment has been classified for the purpose of this paper as a familial factor, though it is important to note that not all maltreatment is perpetrated by a family member. The federal Child Abuse Prevention and Treatment Act (CAPTA) defines maltreatment as child abuse or neglect, which encompasses any act or lack of an act by a child's caretaker that results in physical or emotional harm [28]. Childhood maltreatment, including physical abuse and neglect, has been linked to increased risk for adolescent substance use, with one study reporting 29% of children who experienced maltreatment participating in some level of substance use and another reporting 16% of maltreated children abusing substances [29, 30].

### 3.2. Physical and Sexual Abuse

In most states, the legal definition of physical child abuse entails any act that causes a child to experience physical harm that is not accidental [31]. The effects of physical and sexual abuse, specifically, on adolescent behaviors regarding substance use have been examined, with researchers consistently reporting a statistically significant relationship between physical or sexual abuse and adolescent use of nicotine, marijuana, and alcohol [32–34]. There is also some evidence that higher levels of illicit drug use, including cocaine, heroin, and barbiturates, are associated with physical and sexual abuse [34]. Being a victim of physical or sexual assault increases the risk of an adolescent getting involved with substance use from two to four times [29, 33–35]. However, different studies have shown varying specific results regarding which type of abuse is the strongest contributor, with some reporting a higher risk associated with sexual abuse, while others report a higher risk associated with physical abuse [29, 34]. Posttraumatic stress disorder (PTSD) is also associated with increased likelihood of developing a substance use disorder, particularly with marijuana or hard drugs (including LSD, cocaine, heroin, inhalants, and nonmedical prescription drugs) [33]. This increased risk may be a result of the fact that trauma that typically leads to PTSD is highly stressful and may lead PTSD sufferers to cope with intense stress through substance use [33].

Males are more likely to be physically abused, whereas females are generally more likely to be sexually abused [36]. However, generally speaking, gender differences with regard to substance use vary widely across the literature. Age, though, shows consistent patterns, with older adolescents participating in substance use more often than their younger counterparts, with risk increasing each year from ages 10 to 17 [29, 30, 33, 34]. One review of thirty-five studies indicated that most findings consistently show that childhood maltreatment is a risk factor for earlier onset of substance use [32]. This may be because victims of maltreatment use drugs and alcohol as coping mechanisms rather than purely for social reasons. Thus, their onset is less dependent on the time other adolescents begin to use substances [32].

The association between being a victim of physical or sexual abuse during childhood and adolescent substance use may be linked to the effects of stress on the brain and, specifically, the amygdala [37, 38]. The amygdala is responsible for transmitting emotional information to the body based on memory when responding to stressful situations [37]. When stress, such as that resulting from abuse, arises, the amygdala is overstimulated and excess dopamine is produced as a result, thus suppressing the function of the prefrontal cortex [37, 39]. This cascade of events can lead to limited functions related to attention and learning. Likewise, susceptibility to paranoia has been linked to PTSD [37, 39]. As has been discussed previously, increases in dopamine levels also play a role in addiction to drugs such as opiates, nicotine, ethanol, and cocaine [25, 37].

### 3.3. Emotional Abuse

According to a legal definition, emotional child abuse encompasses a situation whereby the child's “intellectual or psychological functioning or development” is hindered [31]. Research shows that experiencing emotional abuse can lead to increased risk for adolescent substance use, though it does not have as much influence as experiencing physical or sexual abuse [34, 40]. It has also been found that witnessing violence can increase an adolescent's risk for developing a substance use disorder with alcohol, cigarettes, marijuana, or hard drugs by as much as two to three times [33, 35, 40]. This is likely because witnessing violence creates great stress, especially in the case of a child witnessing domestic violence [33]. Therefore, substance use becomes a coping mechanism [33]. It has also been speculated that, in some cases, substance use may precede witnessing violence because such acts of violence may occur within the context of a delinquent peer group where substance use is prevalent [33]. However, there is comparatively little literature that focuses on emotional abuse, including witnessing violence, and its relationship to adolescent substance use and abuse [32].

### 3.4. Neglect

A legal definition of child neglect includes any situation where a child's caregiver does not provide adequate living necessities, including protection, clothing, health care, and/or food [31]. Studies have consistently shown that victims of neglect are at increased risk for substance use [41–43]. Additional research has begun to explore the effects of child neglect on adolescent brain development. Because children in adolescence are undergoing developmental changes, neglect during this period can have long-term effects [41]. It is difficult to study the ramifications of neglect on the brain because of the existence of other contributing factors, such as domestic violence, socioeconomic status, and prenatal exposure to substances [39].

It is also more likely that females' relationship with their parents or conflict within the home will be linked to their choice to use substances than males' [44–46]. This may be because many females respond to stress (as may occur in a negative family environment) by avoiding coping with a situation and increasing attentiveness to emotions, which can heighten depression and lead to substance use, whereas males are often more directly confrontational [47]. These styles of coping may be a result of sociallydefined parameters of gender expectations [47].

### 3.5. Social Risk Factors

Social factors that contribute to increased risk for adolescent substance use include deviant peer relationships, popularity, bullying, and association with gangs. Social influences and familial influences are often present simultaneously. This interaction creates a complex system of risk factors that predicts adolescent substance use, which is important to take into consideration.

### 3.6. Deviant Peer Relationships

The influence of peers on adolescent substance use often exists in the form of deviant peer relationships, wherein an adolescent associates with a group of people who use substances, or in the form of perceived popularity [48–53]. Research has shown that deviant peer relationships are positively associated with adolescent substance use [51, 53]. It is possible that a shared inclination to use drugs and alcohol attracts deviant individuals to form peer groups or that, in order to gain social standing or join a group, individuals are motivated to use substances and thus form a deviant peer group [52, 54].

Entry into deviant peer groups has also been shown to be significantly associated with negative parent-child relationships, which can cause adolescents to seek deviant connections in their social sphere [51]. Conversely, parental involvement and respect for parents have been negatively associated with substance use [52]. This is consistent with the aforementioned findings regarding positive parent-child relationships as a protective factor [29, 42, 44, 45]. This is an example of a way in which factors from familial and social spheres may work for or against each other in leading to adolescent substance use. Some researches have also found that adolescents who grow up in unstable community environments (defined to include lower levels of employment and less access to resources) are actually less susceptible to deviant peer influences [50]. This may be because privileged adolescents may not be exposed to substance use except via peers, whereas underprivileged adolescents face more risk factors, and thus peer influence decreases comparatively [50]. It may also be a result of lower perception of risk of mild experimentation with substances within privileged communities [50].

### 3.7. Peer Pressure and Popularity

Similarly, peer pressure and perceived popularity have been shown to be associated with increased risk for adolescent substance use [48, 49, 52, 55]. Specifically, when adolescents believe that their popularity within a peer group increases with the use of substances, they are more likely to participate in such substance use [48, 49]. Adolescents who self-identify as popular have shown to have increased prevalence of substance use when compared to adolescents who do not identify this way [49]. There may also be a greater correlation between substance use and self-identification of popularity than between substance use and popularity as assessed by peers [49]. Though research into specific types of social motivation is limited, one study revealed that adolescents who seek to be the leader of a group or to stand out above others are more inclined to smoke cigarettes, which can be perceived as an association with maturity, whereas those who seek to be accepted by a group are more inclined towards alcohol use, which is perceived as a communal activity [48]. Boys may also be more likely to engage in smoking to improve their social image, whereas girls more often do so as a form of stress relief [35].

Much of the literature regarding the influences of peer relationships on adolescent substance use focuses primarily on alcohol and cigarette use [48, 52, 53]. Though these areas are important to address, it will be necessary for future research to also focus specifically on marijuana and synthetic marijuana use and prescription drug abuse.

### 3.8. Bullying

The National Institutes of Health define bullying as a series of interactions whereby a group or individual verbally or physically assaults a victim who is perceived to be weaker [56]. All adolescents who participate in bullying, whether they are the perpetrator, the victim, or a combination of both roles, have been shown to have increased risk of mental health disorders and psychosocial problems when compared with those who do not participate [57, 58]. Some research shows that females are more likely to be bullied via verbal attacks and gossip than males, who are usually physically bullied [57]. Males also participate in all roles of bullying at a higher level than females [57, 59].

Research has revealed that playing the role of the bully has been positively associated with increased alcohol use [57, 58]. Interestingly, being a victim of bullying has an inverse association with alcohol use [57, 58]. However, those studies also indicate that victimization is positively associated with other forms of substance use, including marijuana, inhalant, and hard drug use [57, 58]. This is consistent with another study, which found that victims of bullying were more likely to engage in substance use than uninvolved youth [60]. Adolescents who fill the role of both the perpetrator and victim tend to have the highest susceptibility to mental disorders, such as depression and anxiety, though it is not clear whether mental disorders precede bullying or vice versa [58]. The effects of bullying on mental health of participants have shown to be similar among males and females [58].

### 3.9. Gang Affiliation

A legal definition of gangs is a group of three or more people that is characterized by criminal behavior [61]. The literature reveals a significant positive association between gang affiliation and substance use, which has shown to exceed the influence of typical deviant peer groups [62–66]. Specifically, higher rates of alcohol and marijuana use have been reported among gang members than among those who are affiliated with a group of deviant peers [63]. Gangs promote the cycle of substance use, as the appeal of delinquent behavior can attract adolescents to a gang, and, once membership is established, participation in the gang can foster further deviant behaviors and substance use [63].

Familial factors have also been shown to have influence on gang involvement. Risk of substance use as facilitated by involvement with a gang has been shown to decrease in the presence of positive parent-child relationships and authoritative behavioral parenting [64, 65, 67]. The literature often refers to positive familial environment as a protective factor that moderates adolescent substance use via gang involvement [64, 67]. There is some evidence that cultural values of specific ethnic groups can also act as moderating factors or risk factors for adolescent substance use [65, 67].

### 3.10. Individual Risk Factors

Though many risk factors for adolescent substance abuse and dependence are external, there are some individual factors that can contribute to the risk of developing a substance use disorder. Within the literature, two commonly discussed individual risk factors are attention deficit hyperactivity disorder (ADHD) and depression [68, 69]. Likewise, individuals who are diagnosed with posttraumatic stress disorder (PTSD) or mental illness are at greater risk for adolescent substance abuse. Individual sexual orientation and ethnicity, as contributing factors, also appear in the literature, though findings are generally less conclusive.

### 3.11. Attention Deficit Hyperactivity Disorder (ADHD)

Attention deficit hyperactivity disorder (ADHD) is defined by either sustained inattention, characterized in part by forgetfulness and distractedness, or ongoing hyperactivity-impulsivity [68]. In this definition, hyperactivity includes fidgeting and continuously moving, and impulsivity is characterized by interruptions and inability to wait [68]. The DSM-IV estimates that the prevalence of ADHD among school-aged children (from 5 to 17 years old) is approximately 3–5% [68]. However, the CDC reported that, in 2007, 13.2% of male children and 5.3% of female children between the age of 4 and 17 had been diagnosed with ADHD [69]. Furthermore, the CDC has also found that, between 1997 and 2007, the rate of diagnosis of ADHD among children aged from 4 to 17 increased between 3% and 5% each year [69]. It is important to note that this may reflect an increase in reporting statistics or inaccurate diagnoses rather than an increase in prevalence in the past decade.

Several studies, including a meta-analysis of thirteen studies, have indicated that childhood ADHD leads to increased risk of developing a substance use disorder during adolescence or adulthood [70–72]. Specifically, children with ADHD have an increased chance of substance use, with the increased likelihood ranging from 1.47 to 3 times, where the former was based on the development of a substance use disorder and the latter on lifetime use of an illicit drug other than marijuana [71, 72]. Furthermore, the aforementioned meta-analysis concluded that ADHD can specifically lead to increased risk for alcohol or nicotine abuse [70]. However, the meta-analysis of the results regarding the link between marijuana use and ADHD was inconclusive [70].

Researchers have also questioned whether drugs used to treat ADHD (stimulants) may also increase the likelihood of adolescents developing a substance use disorder [71, 73, 74]. However, studies have generally found that stimulant drugs used as medications for the treatment of ADHD do not increase the likelihood that an adolescent will develop substance abuse or dependence [71, 73, 74]. Research, including a meta-analysis of six studies, has revealed that stimulant drugs prescribed for the treatment of ADHD may in fact reduce the risk of developing a substance use disorder by as much as 50% [71, 74].

### 3.12. Depression

The term depression encompasses feelings of sadness, pain, gloom, or anger. Clinical depression specifically refers to situations wherein a person's depressive feelings interrupt their daily life [75]. Depression has been shown to be linked to genetics and may also result from stressors such as parental divorce, parental substance abuse, depression of a family member, or feelings of inadequacy [76–78]. These stressors can lead to feelings of sadness, which some adolescents have reported to be a motivator for them in deciding to begin substance use [78]. This form of “self-medication” is common among adolescents who may not be identified as clinically depressed, yet still suffer from some form of depression [78].

Comorbidity of depression and substance use disorders are common among adolescents, and research has found that these outcomes are linked with each other [77–80]. There is some indication that depressed adolescents may be at higher risk for developing a substance use disorder at an earlier age after the onset of substance use [79]. Furthermore, one study revealed that depressed boys are twelve times as likely to become dependent on alcohol as boys not suffering from depression and that depressed girls are four times as likely to become dependent on alcohol as girls who do not suffer from depression [80]. Additional research has found that the association between depression and alcohol use is stronger among boys, whereas the association between depression and nicotine use is stronger among girls [81].

Several studies have indicated that depression is linked to the brain's reward system, which is known to be affected by the release of dopamine [18, 79, 82–84]. Deficiency of dopamine in the brain, which may be associated with depression, may lead a person to seek other sources for a dopamine fix [82]. As has been discussed, substance use is often associated with increases in dopamine [23, 24]. This may provide some insight into whether depression generally precedes substance abuse and dependence or vice versa. It is possible that some types of substance use cause deficiency in natural production of dopamine, thus leading to depression [25, 82]. It is also possible that depression causes a deficiency in dopamine, which can be alleviated by using substances [82].

The majority of studies have found that depression begins before the onset of substance use, rather than substance use being a precursor to depression [77, 78, 85]. This sequence indicates that the initial lack of dopamine may precede substance use. This is consistent with the idea that feelings of sadness and pain experienced during depression may lead adolescents to seek relief in the form of substance use [78].

## 4. Conclusions

This paper has addressed some prevalent familial, social, and individual risk factors for adolescent substance use. Through the course of this paper, several areas that may require further research have become apparent. First, though some data exist regarding the effects of emotional abuse on adolescent substance use, the strength of research is lacking when compared to that of physical and sexual abuse. Though physical and sexual abuse have been more directly linked to risk for substance use, the effects of emotional abuse (including witnessing violence) should not be overlooked. Secondly, much of the literature focuses exclusively on factors leading to the use of cigarettes and alcohol, especially when discussing peer influences [48, 52, 53]. However, because rates of marijuana use, synthetic marijuana use, and prescription drug abuse are increasing, it will be critical to focus research specifically on these areas in addition to alcohol and tobacco use, which are both on the decline among adolescents in the United States [2]. Finally, though there is value in national samples of data, there is a lack of research pertaining specifically to subregions. Localized studies, especially related to demographic factors, may be more effective in generating results that are specific to particular areas and thus may be more useful in generating and assessing local prevention and intervention efforts.
